# EHMTI-0117. CGRP infusion in awake rats does not increase expression of immediate early genes, c-fos and zif268, in the trigeminal nucleus caudalis

**DOI:** 10.1186/1129-2377-15-S1-A1

**Published:** 2014-09-18

**Authors:** DK Bhatt, R Ramachandran, SLT Christensen, S Gupta, I Jansen-Olesen, J Olesen

**Affiliations:** 1 Department of Neurology, Glostrup Hospital Faculty of Health and Medical Sciences University of Copenhagen, Glostrup, Denmark

## Background and aims

Calcitonin gene-related peptide (CGRP) and glyceryl trinitrate (GTN) infusion in migraineurs provokes headache resembling spontaneous migraine. GTN infusion model was transformed to rats causing expression of protein markers as a surrogate for headache. We hypothesized that CGRP infusion in awake rats would increase molecular markers of neuronal activation in migraine relevant tissues.

## Methods

CGRP was infused intravenously in freely moving rats. c-fos mRNA in trigeminal nucleus caudalis (TNC) was analyzed by qPCR at different time points after CGRP and saline infusion. c-Fos and Zif268 stained nuclei were counted in the TNC. c-Fos-positive nuclei were also counted in the nucleus tractus solitaries (NTS) and caudal ventrolateral medulla (CVLM), integrative sites in the brain stem for processing cardiovascular signals. Protein expression of phosphorylated-extracellular signal-regulated kinase (p-ERK), p-CREB and c-Fos was analysed in the dura mater, trigeminal ganglion and TNC samples using western blot.

## Results

CGRP infusion caused a fall in blood pressure, and activated c-Fos in the NTS and CVLM in the brain stem. After 30 min of CGRP infusion, an increase in p-ERK was observed in the dura mater. But no activation of the neuronal activation markers, c-Fos and Zif269, was observed in the TNC.

## Conclusion

CGRP infusion caused early activation of p-ERK in the dura mater but it did not increase immediate early genes in the TNC. Thus, systemic CGRP infusion substantially acts outside blood brain barrier and the peripheral activation doses not lead to the activation of second-order trigeminal neurons.

No conflict of interest.

